# Deployment of Attention on Handshakes

**DOI:** 10.3389/fpsyg.2016.00681

**Published:** 2016-05-09

**Authors:** Mowei Shen, Jun Yin, Xiaowei Ding, Rende Shui, Jifan Zhou

**Affiliations:** Department of Psychology and Behavioral Sciences, Zhejiang UniversityHangzhou, China

**Keywords:** visual organization, attention, social relationship, attentional unit, social cognition

## Abstract

Understanding the social structures between objects, organizing, and selecting them accordingly, is fundamental to social cognition. We report an example that demonstrates the object association learned from social interactions could impact visual attention. Particularly, when two hands approach each other to perform a handshake, they tend to be attended to as a unit because of the cooperative relationship exhibited in the action: even a cue presented on a non-target hand may facilitate a response to the targets that appear on the non-cued hand (Experiment 1), indicating that attentional shift between two hands was facilitated; furthermore, the response to a target on one hand is significantly impaired by a distractor on the other hand (Experiment 2), implying that it is difficult to selectively confine attention to a single hand. These effects were dependent on the existence of the hands when cue and target appeared (Experiment 3); neither perceptual familiarity, or physical fit can explain all the attention effects (Experiment 4). These results have bearings on the perceptual root of social cognition.

## Introduction

Social relation between objects (or agency) has significant meanings for mankind. People’s interactions assign social properties to the structure of agents; for example, people who help each other have cooperative relationships. Understanding social structure and organizing agents accordingly is important in daily life, because we frequently need to pool individuals who cooperate with each other into groups and distinguish allies from opponents. Since social relationship defines the social units we process during social cognition, the mental system should organize and select information according to social relationship at some early stage of processing, before high-level social cognition. The human visual system is equipped with remarkably efficient computation modules to extract social properties from low-level visual features, including the impression of animacy, others’ goals and intentions (Heider and Simmel, 1944; Dittrich and Lea, 1994; Scholl and Tremoulet, 2000; Schlottmann et al., 2006; Gao et al., 2009, 2010). And those perceived social information is able to affect lower-level perceptual processing. For example, our previous research demonstrated that social cues affect the causal perception of physical events (Zhou et al., 2012), and social relationship shapes the visual organization of objects that dynamically interact with each other (Yin et al., 2013). Thus, noting that social structure is important for humans to organize the observed word, we speculated that social information may modulate attention in enabling the social-based deployment of processing resource.

Despite the importance of social structures, the traditional research about attention majorly focused on physical structures, which are defined by physical relationships between objects, such as proximity, symmetry, and continuity. According to those structures, the visual system group the visual elements into processing units at different levels, such as figure-ground segregation, object forming, and perceptual grouping (Wertheimer, 1923; Marr, 1982; Treisman, 1986; Scholl, 2001; Palmer, 2003), based on some fundamental perceptual principles, such as Gestalt laws (Sternberg, 2003). It enables us to see a well-structured visual world rather than discrete visual elements. Attention deployment is guided by such physical structures; a great example is the well-studied “same-object advantage” effect (Duncan and Duncan, 1984; Kahneman et al., 1992; Egly et al., 1994; Moore et al., 1998; Flombaum and Scholl, 2006), which shows that attention automatically spreads within an object defined by real or illusory contours. In addition to objects, a similar effect was suggested to occur for perceptual groups (Driver et al., 2001; Scholl, 2001). That is, attention automatically spreads within a group, and it is difficult to attend to a single object in a group without also selecting the other objects.

Indeed, recent studies revealed that social information also has a profound impact on attention deployment. Joint attention (Frischen et al., 2007) is probably the best example of that: participants’ performance on an object detection task was facilitated when a preceding face cue gazed toward the location of the target. This gaze cueing effect occurs rapidly with very brief presentation of gaze cues (Friesen and Kingstone, 1998), and occurs such that the gaze cue does not necessarily predict the direction of, or even predicts the direction opposite to, the target location (Driver et al., 1999; Friesen et al., 2004). Moreover, the gaze-following attention shift may occur without the awareness of the presentation of gaze cues (Sato et al., 2007). These findings suggest that the visual system automatically aligns attention with that of others based on the received social information—other’s gaze direction, which signals their locus of attention.

The gaze cueing effect demonstrates that attention deployment is modulated by the social relationship defined by the interaction between other people and objects. In addition to gaze, other body parts that are involved in social interaction, such as hands, also affect attention. For instance, attention prioritizes stimuli in near-hand space (Reed et al., 2006), and the attentional system treat the surface of one’s own hands differently from other surface, therefore shifting attention from hands to other surface (including other hands) is costly (Taylor and Witt, 2014; Taylor et al., 2015). These hand-based effects on attention, together with the visuomotor priming caused by hands (e.g., Bruzzo et al., 2008; Liuzza et al., 2012), facilitate manual action and help people understand the relationships in social interactions. Our previous research also showed that social interaction between objects (or agents) affects attention deployment (Yin et al., 2013). In this study, a chasing motion including two predators and one prey was presented to participants. In the condition that predators cooperated to chase the prey, the predators were perceived as a group, such that the exogenous attentional cue appearing on one of the two predators could facilitate the response to the target presented on the other (uncued) predator. This automatic attention spreading did not occur when the predators acted in a competitive manner to chase the prey suggesting that the social relationship (cooperative/competitive relationship in this case) obtained from the dynamic motion display can guide our attention deployment.

Based on those findings, the current study aimed to generalize the social-based attention effect to socially related objects acquired from social learning. Baron-Cohen (1995) suggested that the behavior of joint attention is performed by an innate module attuned to the visual appearance of the eyes. Furthermore, the ability to perceive causality and animacy (the foundation of detecting cooperative/competitive chasing) from motion display is considered to be innate (see Scholl and Tremoulet, 2000 for a review). Thus, social-based attention effects may occur on only stimuli that are processed by innate domain-specific modules that developed through evolution to adapt to social life. However, neither this hypothesis nor its alternative hypotheses have been directly examined; thus, it is still unclear exactly what type of social information is able to modulate attention deployment. Here, we tested whether such effects occur on learned socially related objects, in order to add a new type of social-based attention deployment to the known body of knowledge of attention.

The current study used a handshake scene, in which two hands approach each other to perform a handshake, to examine the attention effect induced by socially related objects (i.e., the two hands). The handshake scene was selected because of the following considerations: (1) the handshake is a greeting gesture learned in social interaction, not an innate behavior; not even a universal behavior. (2) Handshakes symbolize friendship, agreement, congratulatory sentiments, and hence provide social information about cooperative relationship between individuals. Thus, the hands involved in the handshake scenes may be treated as a social unit, leading to modulation on attention deployment. (3) Handshakes are so common in daily social interactions that people have learned the association of the two hands going to perform a handshake; thus, the social meaning is easily understood and more likely to instantly impact attention deployment. Compared to physical cues, information about the social relationship of objects are not usually obvious or instantly available; thus, if the social structure of scenes impact attention, it would most likely occur in visual scenes in which the social relationship of objects has been well learned in our daily life. (4) The motion involved in handshakes is relatively simple; thus, handshakes can be manipulated and controlled in laboratory settings.

If the socially related objects (i.e., the two hands) were treated as a unit during attentional processes, similar to perceptual units defined by physical cues, then we could hypothesize that (1) when attention is cued to one hand, the response to a target presented on the other hand would be quicker because the attentional shift from the cued hand to the other is facilitated; and (2) a distractor presented on one hand would significantly interfere with a target presented on the other hand because it would be difficult to selectively confine attention to just one hand. These two hypotheses were tested in the following experiments.

## Experiment 1

Experiment 1 explored whether two hands that are approaching to perform a handshake would facilitate attentional shift between two hands. This social-based attention effect was examined using a cueing task similar to that used by Moore et al. (1998). In this task, participants are presented with a display where two hands (one showing its palm and the other showing its back) are approaching each other, creating the impression that they will perform a handshake. Subsequently, the hands return to their initial positions, and a target randomly appears on one hand. Before the target appears, one of the hands is cued by the onset of a white square. In most trials, the cue appears on the same hand as that where the target will be presented (*valid* trials); in the remaining trials, the cue appears on the other hand (*invalid* trials). Typically, the response to the target is faster when the cue is valid than when it is invalid. However, if the two hands are treated as an attentional unit bound by their social relationship, then attentional shift between the cued hand to the non-cued hand, decreasing the RT difference between the valid and invalid trials. Consequently, the cueing effect should be weakened when the hands are approaching to perform a handshake. Three control conditions were designed as follows: the *both-back* condition, in which the backs of both of the hands were shown; the *both-palm* condition, in which the palms of both hands were shown; and the *reversed-hand* condition, in which a palm and the back of a hand were presented, as in the *handshake* condition, except that one of the hands was shown upside-down. In all three of the control conditions, it was impossible to create the impression of a handshake.

### Methods

#### Participants

Students from Zhejiang University aged between 18 and 26 years (∼21 on average) were recruited to participate in this and following experiments. All were right-handed, had normal or corrected-to-normal vision and were naïve about the purpose of the experiments. The participants received financial rewards after completing the experiment. The experimental protocol was approved by the Institutional Review Board at the Department of Psychology and Behavioral Sciences, Zhejiang University; participants gave written informed consent prior to their participation.

Twenty naïve students (11 men and 9 women) participated in Experiment 1. To ensure adequate power, the sample size was determined by a power analysis based on predicted effect size using G*power 3 (Faul et al., 2007, 2009). Based on the results of our pilot studies, we predicted a medium effect size (*f*^2^ = 0.15, according to Cohen, 1988) for our experimental design. With the alpha level set at 0.01, the suggested sample size was approximately 20 individuals. The sample sizes of the following experiments were determined by the same rule.

#### Design and Procedure

The participants were tested individually in a darkened room while seated approximately 60 cm from the screen. All displays were presented on a gray background on a Samsung 19^′′^ CRT monitor, which subtended approximately 28.3° in visual angle.

**FIGURE 1:**
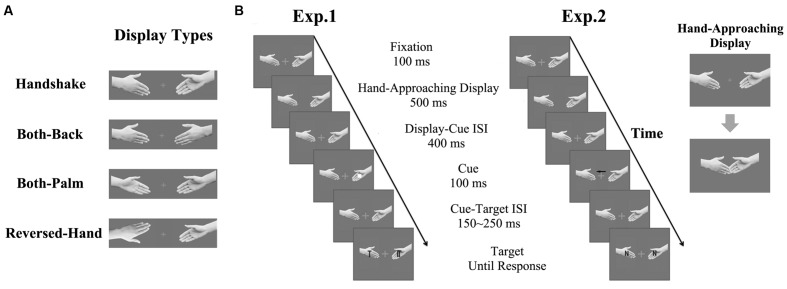
**Depiction of the four hand-approaching display conditions **(A)** and the procedure of Experiments 1 and 2 (B).** The four hand-approaching display conditions were as follows: (1) the palm of one hand and the back of the other were displayed such that they were prepared for a handshake (the *handshake* condition); (2) the backs of both hands were displayed (the *both-back* condition); (3) the palms of both hands were displayed (the *both-palm* condition); and (4) the palm of one hand and the back of the other were displayed, but one of the hands was upside-down (the *reversed-hand* condition). The beginning and the end of the hand-approaching display were showed at the right. In Experiment 1, the task was to report whether a *T* or an *L* was presented on one of the hands; the cue was valid or invalid (here, the invalid condition is shown). In Experiment 2, the task was to report whether an *N* or an *X* appeared on the hand indicated by the arrow; the character presented on the other hand was compatible or incompatible with the target (here, the compatible condition is shown).

Each trial began with a separate presentation of two hands; these presentations were separated by a white fixation cross (0.5° × 0.5°). The fixation was presented at the center of the screen; the visual angle between the fixation and each fingertip was 2°. The appearance of the two hands was in accordance with one of the four hand-approaching display types shown in **Figure 1A**. After being stationary for 100 ms, the hands began to approach each other until the images overlapped by 0.7°. Following this display, the overlapped hands vanished and immediately reappeared at their initial locations. After an interval of 400 ms, a cue (a 1.2° × 1.2° white square) was presented at the center of one hand for 100 ms. Following a variable interstimulus interval (ISI; 150–250 ms), a target and a distractor appeared on the two hands. The target was a *T* or an *L*, whereas the distractor was one of three *T–L* hybrid characters, which were identical to those used by Moore et al. (1998). These characters subtended 0.73° × 0.73° each and were randomly oriented at one of four orientations (0, 90, 180, or 270°). The participants were asked to report whether the target was a *T* or an *L* as quickly as possible by pressing the right or left buttons on a standard keyboard. They were explicitly informed that the displays of hand movement were irrelevant to the task and could, therefore, be ignored. The target and distractor remained visible until a response was made. The next trial began after a 1.5–2.5-s intertrial interval. If no response was made within 2 s, the next trial began immediately.

Each participant completed 360 trials (90 trials in each of the 4 hand-approaching display conditions). In two-thirds of the trials (i.e., 60 trials in each condition), the cue and target appeared on the same hand; these trials were valid trials. In the remaining one-third of the trials, the cue and target appeared on different hands; these trials were invalid trials. All of the trials were presented in random order.

### Results and Discussion

**FIGURE 2:**
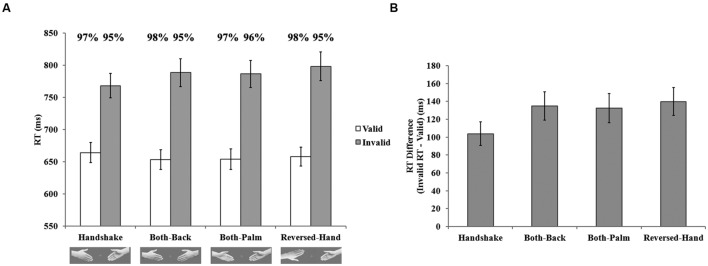
**Results of Experiment 1. (A)** Mean reaction time (RT) varied as a function of hand-approach display and cue validity. Mean accuracy rates for every condition was shown above the corresponding bars. **(B)** The mean RT difference between invalid and valid trials varied according to the hand-approach display. Error bars indicate standard errors.

The mean RTs for correct responses in the four conditions are shown in **Figure 2A**. The RT data were submitted to a 2 (validity) × 4 (hand-approaching display) repeated-measures ANOVA. The main effect of validity was significant, *F*(1,19) = 95.80, *p* < 0.001, $\eta_{p}^{2}$ = 0.83, but that of hand-approaching display was not significant, *F*(3,57) = 1.84, *p* > 0.05, $\eta_{p}^{2}$ = 0.09. The interaction between validity and approach display was significant, *F*(3,57) = 3.86, *p* < 0.05, $\eta_{p}^{2}$ = 0.17, indicating that the cueing effect varied across the hand-approaching display conditions. **Figure 2B** shows the mean RT differences between the valid and invalid trials, which reflect the facilitation induced by the attention cue. A *post hoc* LSD test revealed that the mean RT difference in the handshake condition was significantly smaller than that in the control conditions (*handshake* vs. *both-back*, *p* < 0.01; *handshake* vs. *both-palm*, *p* < 0.05; *handshake* vs. *reversed-hand*, *p* < 0.001). Thus, the cueing effect was weakened in the handshake condition, compared to each of the three different control conditions. As shown in **Figure 2**, this weakened cueing effect stemmed from both the faster responses in the invalid trials (*handshake* vs. *both-back*, *p* < 0.05; *handshake* vs. *both-palm*, *p* < 0.05; *handshake* vs. *reversed-hand*, *p* < 0.01) and the slower responses in the valid trials (relatively weak, but there was a trend toward significance, *handshake* vs. *both-back*, *p* = 0.06; *handshake* vs. *both-palm*, *p* = 0.06; *handshake* vs. *reversed-hand*, *p* = 0.10) compared to the control conditions.

The same ANOVA was conducted for the accuracy data, revealing significant main effect of validity, *F*(1,19) = 20.31, *p* < 0.001, $\eta_{p}^{2}$ = 0.52. It suggested that the responses were more accurate on valid than invalid trials (97% vs. 95%). No other effect reached statistical significance, *F*s < 2.25, *p* > 0.12. Thus there was no speed-accuracy tradeoff.

The faster responses in the invalid trials indicated that attentional shift from the cued hand to the other hand was facilitated. Therefore, the processing of targets that appeared on the uncued hand was speeded. Meanwhile, the attentional resources allocated to the cued hand were diluted, leading to the slower processing of targets that appeared on the cued hand. The hypothesis that two hands approaching to perform a handshake would be treated as an attentional unit—and, hence, that the cueing effect would be weakened—was supported by the results of Experiment 1.

## Experiment 2

Experiment 2 employed an interference task to investigate whether the handshake display would increase the interference between the two hands. In this task, an arrow is presented in the center of the screen to indicate the hand on which the target will be presented (here, the rate of cue validity was 100%). Then, a target appeared on the cued hand and a distractor concurrently appears on the other hand. The distractor may be the same character as the target (*compatible* trials) or a different character (*incompatible* trials). Typically, participants’ responses to the target are more rapid in compatible than in incompatible trials; this RT difference reflects the interference effect. Much like in Experiment 1, we manipulated the hand-approaching display conditions. We posited that if the two hands were treated as an attentional unit when they seemed poised to perform a handshake, then participants would be unable to selectively confine their attention to a single hand without attending to the other. We thus expected the incompatible distractor to produce greater interference compared to the control conditions, in which the hands were not socially related.

### Methods

Twenty naïve Zhejiang University students (9 men and 11 women) participated and received financial rewards. None of them had participated in Experiment 1.

The hand-approaching displays were identical to those in Experiment 1 (see **Figure 1**). Following the display, an arrow that pointed to the right or left was presented above the fixation. With an ISI of 150–250 ms, a target appeared on the hand where the arrow was pointing, and a distractor was presented on the other hand. The target and the distractor were either an *N* or an *X*. In half of the trials, the target and distractor were compatible (i.e., both *N* or both *X*); in the remaining half of the trials, the target and distractor were incompatible. The participants’ task was to report whether the target was an *N* or an *X* as quickly as possible by pressing the left or right buttons, respectively. The target and distractor remained on the screen until a response was made. A 2 (compatibility: compatible, incompatible) × 4 (hand-approaching display) within-subjects design was used. Each participant completed 560 total trials, with 70 trials in each condition. The trials were presented at random.

### Results and Discussion

**FIGURE 3:**
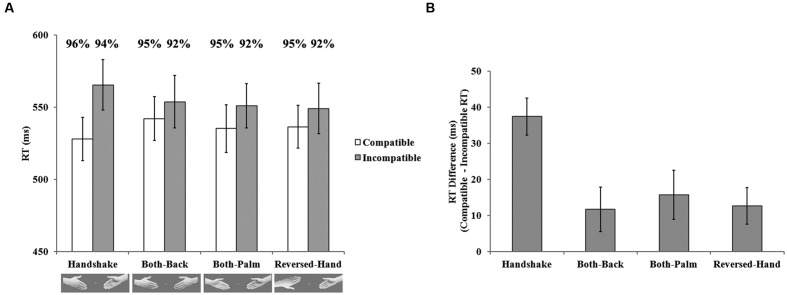
**Results of Experiment 2. (A)** Mean RT varied as a function of hand-approaching display and compatibility. Mean accuracy rates for every condition was shown above the corresponding bars. **(B)** The mean RT difference between compatible and incompatible trials according to the hand-approaching display is shown. Error bars indicate standard errors.

A 2 (compatibility) × 4 (hand-approaching display) repeated-measures ANOVA indicated a significant main effect of compatibility, *F*(1,19) = 21.43, *p* < 0.001, $\eta_{p}^{2}$ = 0.53, but no main effect of hand-approaching display, *F*(3,57) = 1.15, *p* > 0.05, $\eta_{p}^{2}$ = 0.06. The interaction between these two variables was significant, *F*(3,57) = 6.94, *p* < 0.01, $\eta_{p}^{2}$ = 0.27, indicating that the interference effect varied across hand-approaching display conditions. To compare the interference effect across the four hand-approaching displays, we submitted the mean RT difference between the compatible and incompatible trials to a single factor (hand-approaching display) repeated-measures ANOVA with *post hoc* LSD test. The results revealed a significant main effect, *F*(3,57) = 6.89, *p* < 0.01, $\eta_{p}^{2}$ = 0.27, and the interference effect was significantly larger in the handshake condition than in the control conditions, *p*s < 0.05. As shown in **Figure 3**, faster RT in the compatible trials [*handshake* vs. *both-back*, *p* < 0.05; *handshake* vs. *both-palm*, *p* = 0.18 (not significant between these two conditions); *handshake* vs. *reversed-hand*, *p* < 0.01] jointly contributed with slower RT in the incompatible trials (for all paired comparisons, *p* < 0.05) to the largest interference effect in the handshake condition.

The same ANOVA was conducted for the accuracy data. No effect was significant, *F*s < 2.59, *p* > 0.12, suggesting no speed-accuracy tradeoff.

This result implied that the distractor shared attentional resources with the target, leading to a facilitation of responses with a compatible distractor and a delayed response with an incompatible distractor.

## Experiment 3

Experiment 3 was conducted to examine what would happen when hands were not presented after the hand-approaching display. Our hypothesis to be tested was that social information modulates the attention deployment on the related objects, if it was true, then the effect should be specific to the two hands – nothing would happen if the hands disappeared, because the objects to be attend to went away in this case. Otherwise, if the hand-approaching display induced a general attention effect, such as increasing the breadth of attention or facilitating the attentional shift between all objects, and the effect lasted for a certain time to affect the subsequent task, it would still occur when hands were removed after the approaching action. Because the displays in the previous experiments all contained two hands, it was difficult to distinguish whether the observed results occurred due to a general effect affecting all objects. In Experiment 3, both the hands were removed *after* the hand-approaching display, and the targets and distractors were presented directly onto the background, to test whether the effects found in Experiments 1 and 2 were dependent on the image of the hands.

### Methods

This experiment involved two sub-experiments. One sub-experiment used the cueing task that served as the control situation in Experiment 1; the other used the interference task that served as the control of Experiment 2. Each of the sub-experiments included 20 participants (10 men and 10 women in each sub-experiment).

The sub-experiment with the cueing task was similar to Experiment 1; however, we added a condition in which the hands disappeared prior to target onset. While the procedure was identical to Experiment 1 in the hands-present condition, the hands disappeared following the cue offset in the hands-disappear condition. Because adding the hands-disappear condition doubled the length of the experiment, we removed the both-back condition and the both-palm condition, which were not significantly different from the reversed-hand condition in the previous experiments. Such changes allowed us to maintain an appropriate experimental duration. This sub-experiment employed a 2 (hands status: hands-present, hands-disappear) × 2 (hand-approaching display: handshake, reversed-hand) × 2 (validity: valid, invalid) within-subjects design. The total number of trials, presented in a random order, was 360.

Similarly, the sub-experiment with the interference task had an additional hands-disappear condition, in which the hands disappeared after the cue offset. Again, we removed the both-back condition and the both-palm condition. Thus, this sub-experiment used a 2 (hands status: hands-present, hands-disappear) × 2 (hand-approaching display: handshake, reversed-hand) × 2 (compatibility: compatible, incompatible) within-subjects design. Each participant completed 240 trials (30 in each condition).

### Results and Discussion

#### Cueing Task

**FIGURE 4:**
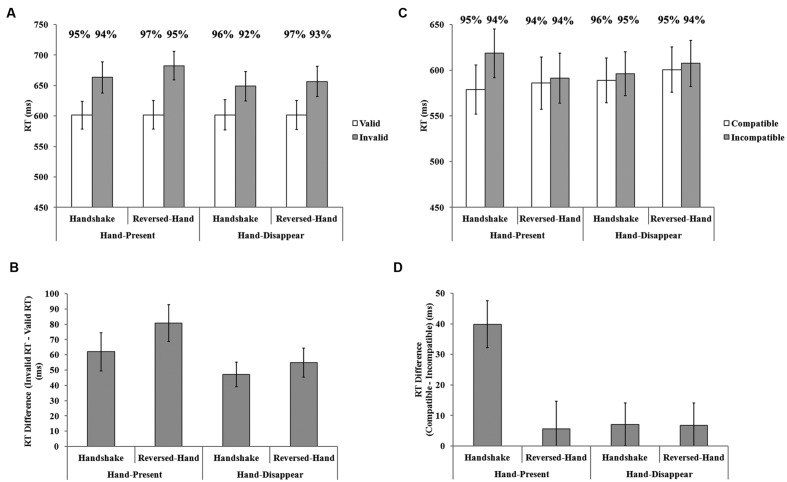
**Results of Experiment 3. (A)** and **(B)** respectively, depict the mean RT and RT difference between the invalid and valid trials for the cueing task. **(C)** and **(D)** respectively, depict the mean RT and RT difference between the compatible and incompatible trials for the interference task. Error bars indicate standard errors. Mean accuracy rates for every condition was shown above the corresponding bars.

**Figure 4A** shows the mean RTs for correct trials in the four conditions of the cueing task. To examine the social-information-induced attention effect in different hands status (hands-present/hands-disappear), we ran a 2 (hands status) × 2 (hand-approaching display) × 2 (validity) repeated-measures ANOVA for the mean RTs in correct trials. The result showed significant main effects of hand-approaching display, *F*(1,19) = 9.14, *p* < 0.01, $\eta_{p}^{2}$ = 0.33, and validity, *F*(1,19) = 42.81, *p* < 0.001, $\eta_{p}^{2}$ = 0.69. The interaction between hands status and validity was significant, *F*(1,19) = 8.03, *p* < 0.05, $\eta_{p}^{2}$ = 0.30. And the interaction between hand-approaching display and validity was significant, *F*(1,19) = 5.28, *p* < 0.05, $\eta_{p}^{2}$ = 0.22. Other effects were failed to reach significance, *F*s < 1.33, *p*s > 0.26, including the 3-way interaction, although when hands were present the mean RT difference between valid and invalid trials was smaller in the handshake condition than in the reversed-hand condition (see **Figure 4B**), *t*(19) = 3.37, *p* < 0.01, which replicating the result of Experiment 1; while this difference was not significant when hands were removed, *t*(19) = 0.86, *p* = 0.40. The insignificant 3-way interaction was probably because that the differential RTs were in the same direction for the hands-present and hands-disappear conditions: the handshake display led to smaller cueing effect than the reversed-hand display, in both the hands-present and hands-disappear conditions, although the difference of the cueing effect between handshake and reversed-hand conditions was numerically larger when hands were present (19 ms vs. 8 ms).

The accuracy data were submitted to the same ANOVA. The only significant effect was validity, *F*(1,19) = 10.34, *p* < 0.01, $\eta_{p}^{2}$ = 0.35, indicating that the accuracy was higher when cue was valid than invalid (97% vs. 94%). The interaction between hands status and validity was marginally significant, *F*(1,19) = 3.53, *p* = 0.08, $\eta_{p}^{2}$ = 0.16. Other effects were not significant, *F*s < 2.76, *p*s > 0.11.

Based on the insignificant 3-way interaction of the RT data, it was not able to make a conclusive answer to the question whether the attention effects induced by handshake display dependent on the image of the hands. One possible explanation for that is the handshake display might produce a general effect that facilitating the attention shifts between all objects (probably very weak), which contributed to the effects found in Experiments 1 and 2; but it cannot completely explain the results, since the effect in the hands-disappear conditions is smaller than that in the hands-present condition. Thus, the whole picture would be clearer in combination of the interference effect.

#### Interference Task

**Figure 4C** shows the mean RTs for correct trials in the four conditions of the interference task. For the interference task, the 2 (hands status) × 2 (hand-approaching display) × 2 (compatibility) repeated-measures ANOVA revealed significant main effect of compatibility, *F*(1,19) = 7.57, *p* < 0.05, $\eta_{p}^{2}$ = 0.29. The other two main effects were not significant, *F*s < 0.37, *p*s > 0.55. All the 2-way interactions were significant: hands status × hand-approaching display, *F*(1,19) = 7.44, *p* < 0.05, $\eta_{p}^{2}$ = 0.28; hands status × compatibility, *F*(1,19) = 4.90, *p* < 0.05, $\eta_{p}^{2}$ = 0.21; hand-approaching display × compatibility, *F*(1,19) = 10.55, *p* < 9.01, $\eta_{p}^{2}$ = 0.36. The 3-way interaction was significant, *F*(1,19) = 6.17, *p* < 0.05, $\eta_{p}^{2}$ = 0.25, indicating that hands status and hand-approaching display interact together to impact the interference effect. As shown in **Figure 4D**, when the hands were present, the interference effect in the handshake condition was significantly larger than that in the reversed-hand condition, *t*(19) = 4.11, *p* < 0.001; while this difference is not significant when hands were removed, *t*(19) = 0.03, *p* = 0.97. The ANOVA for accuracy data found no significant effect, *F*s < 2.24, *p*s > 0.15. These results suggested that the handshake display increased the interference effect only when the hands were present.

Thus, the result of this sub-experiment clearly showed that the interference effect induced by the handshake display vanished when hands were removed. The result of the cueing task was consistent, as the weakened cueing effect induced by the handshake display did fade away in the hands-disappear condition, although the interaction failed to reach statistical significance. Combining the results from both sub-experiments, this experiment showed that the effect is transient and dependent on the existence of the two hands, providing further evidence to support our hypothesis that the handshake display modulated the attention deployment on the hands.

## Experiment 4

Before conclusions can be drawn, other alternative accounts must be excluded. In addition to its social meaning, the handshake display used in Experiments 1 and 2 is significantly different from the control conditions in two aspects. The first difference is related to perceptual familiarity. People frequently view handshakes but rarely view the scenes depicted in the control conditions; because two hands approaching each other are typically perceived as a grouped entity in an everyday handshake, the display of two approaching hands may activate the representation of two hands holding together. If this account is accurate, then it is probable that social information is not the key factor in determining the attention effect; rather, it is prior experience (i.e., the learned association between objects, also including learned social relationships) that leads to attending to both of the displayed hands, thus not only socially related objects but all kinds of objects that have learned object associations would produce similar attention effects. The second difference is physical fit. That is, the hands in the handshake condition could lock on to each other but the hands in the both-back and both-palm conditions could not. Although the hands in the reversed-hand condition are capable of grasping each other, this act is not as easy to see as in the handshake condition. Thus, the attention effect might be attributed to the physical fit between the two hands rather than social implications.

To test whether mere perceptual familiarity or physical fit result in the attention effects, we introduced a *hand-cup* condition and a *plug-socket* condition in addition to the handshake and reversed-hand conditions. In the hand-cup condition, one of the two hands in the handshake condition was replaced by a cup, which appeared as if it was going to be caught by the hand. In the plug-socket condition, the hands were replaced by a plug and a matching socket, creating the impression that the plug will be plugged into the socket. Both scenes are generally familiar to participants, and the object associations in these scenes are well learned. For the student participants in the present study, the two new scenes may have been even more familiar than the handshake scene. Participants drank from cups several times each day, and plugged in laptops or cell phones frequently. By contrast, these young students were less likely to view handshake scenes on a daily basis. Moreover, the objects (hand and cup, plug and socket) that appeared in each scene were a clear fit with each other.

Hence, according to the perceptual familiarity and physical fit account, we should observe similar attention effects in these two conditions, because the objects are familiar and fit together well. However, compared to the handshake scene that exhibits a clear social relationship between two men, the hand-cup scene is a much less social situation because it shows an active behavior toward a passive object; and the plug-socket scene shows a non-social relationship, which is determined by their shapes that are physically fit with each other. Thus, if social relationship is the key factor to modulate the deployment of attention, the handshake condition should lead to different results from the other two conditions.

### Method

Again, Experiment 4 involved two sub-experiments with separate cueing and interference tasks. Twenty naïve Zhejiang University students (9 men and 11 women) participated in the cueing version and another 20 students (11 men and 9 women) participated in the interference version.

The procedure of the cueing and interference task was similar to that of Experiments 1 and 2, respectively, except that the both-back and both-palm conditions were replaced by the hand-cup and plug-socket condition. The cup, plug, and socket had the same width as the hand, and their approaching display adopted the same moving pattern: the hand and cup (or, the plug and socket) moved to approach each other until the images overlapped by 0.7°. In the cueing task, a cue was presented at one of the objects (which could be a hand, cup, plug, or socket, depending on the condition), followed by a target and a distractor appeared on the two objects. Again, participants performed a “T-or-L” task as quickly as possible. All the parameters (color, shape, size, and duration, etc.) of the cue and target were identical to those of Experiment 1. In the interference task, a central cue (the same arrow as Experiment 2) was presented after the approaching display. Following the cue, a target appeared on the cued object, and a distractor appeared on the uncued object. The same “N-or-X” task was performed. All other experimental details of the interference task were identical to those of Experiments 2.

The cueing task sub-experiment employed a 2 (validity) × 4 (hand-approaching display: *handshake*, *hand-cup*, *plug-socket*, and *reversed hand*) within-subjects design, with 90 trials (in which 60 trials were cue-valid, and 30 trials were cue-invalid) in each of the 4 hand-approaching display conditions, resulting in 360 trials in total. The interference task sub-experiment employed a 2 (compatibility) × 4 (hand-approaching display) within-subjects design, with 70 trials in each combined condition, resulting in 560 total trials in total. All of the trials were presented in random order.

### Results and Discussion

#### Cueing Task

**FIGURE 5:**
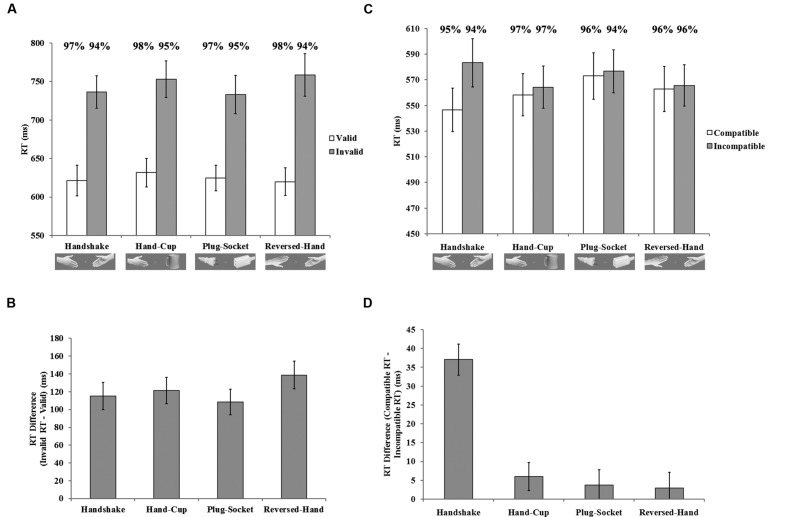
**Results of Experiment 4.** Results of Experiment 3. **(A)** and **(B)** respectively, depict the mean RT and RT difference between the invalid and valid trials for the cueing task. **(C)** and **(D)** respectively, depict the mean RT and RT difference between the compatible and incompatible trials for the interference task. Error bars indicate standard errors. Mean accuracy rates for every condition was shown above the corresponding bars.

**Figure 5** (left) shows the mean RTs for correct responses in the cueing task. A 2 (validity) × 4 (hand-approaching display) repeated-measures ANOVA revealed significant main effects of validity, *F*(3,57) = 105.39, *p* < 0.001, $\eta_{p}^{2}$ = 0.85, and hand-approaching display, *F*(3,57) = 3.99, *p* < 0.05, $\eta_{p}^{2}$ = 0.17, indicating that RT varied with cue validity and display type. However, the interactive effect failed to reach statistical significance, *F*(3,57) = 2.49, *p* = 0.09, $\eta_{p}^{2}$ = 0.12, suggesting that the cueing effect did not vary much across the hand-approaching display conditions. The cueing effect, namely the mean RT difference between valid and invalid trials, for the handshake condition (115 ms) was smaller but not significantly so compared to the reverse-hand condition (139 ms), *t*(19) = 1.63, *p* = 0.12. It was also not significantly different from that of the hand-cup (121 ms) and plug-socket (108 ms) display.

The same ANOVA was conducted for the accuracy data, revealing significant main effect of validity, *F*(1,19) = 15.06, *p* < 0.001, $\eta_{p}^{2}$ = 0.44. It suggested that the responses were more accurate on valid than invalid trials (97% vs. 94%). No other effect reached statistical significance, *F*s < 1.54, *p* > 0.22.

Thus, compared to the control conditions, the handshake condition showed nothing special in modulating the cueing effect, because the cueing effect failed to be significantly smaller than the control conditions. That was not the expected result, so we returned to the result of Experiment 1. After comparing the data from the two Experiments, we found that the cueing effect for the reverse-hand condition remained unchanged (140 ms in Experiment 1 and 139 ms in Experiment 4), although the overall RT decreased; on the other hand, the cueing effect for the handshake condition increased slightly (from 104 ms in Experiment 1 to 115 ms in Experiment 4). As for the hand-cup and plug-socket condition, the cueing effect was low. Thus, we speculated that the familiar non-socially paired objects are also able to facilitate attentional shift between the related objects, and that this facilitative effect is not exclusively for socially related objects. Moreover, since the cueing effect is sensitive to variable factors, such as cue validity, perceptual load, and the subjective organization of stimuli (Chen, 1998; He et al., 2004; Ho and Atchley, 2009), the facilitated attentional shift is likely to be attenuated when other frequently paired objects are presented at the same time. Specifically, when there were three different kinds of paired objects in the same experiment, making most of the trials included related objects, the attentional system might switch to an “easy-shift” mode in those trials while treat the only different condition – the reversed-hand condition in a special way. In this case, the handshake condition is treated equally with the hand-cup and plug-socket conditions, therefore diluting the effect induced by the handshake scene, compared to Experiment 1. That is a possible explanation for this experiment failing to obtain a significant smaller cueing effect in the handshake condition than in the reversed-hand condition.

#### Interference Task

**Figure 5** (right) shows the mean RTs for correct responses in the interference task. A 2 (compatibility) × 4 (hand-approaching display) repeated-measures ANOVA revealed a significant main effect of compatibility, *F*(1,19) = 29.60, *p* < 0.001, $\eta_{p}^{2}$ = 0.61. The main effect of hand-approaching display was also significant, *F*(3,57) = 5.75, *p* < 0.01, $\eta_{p}^{2}$ = 0.23, suggesting that RTs varied with the “background” of the target (i.e., responses were slowest with the plug-socket background). The interaction was significant, *F*(3,57) = 18.44, *p* < 0.001, $\eta_{p}^{2}$ = 0.49, indicating that the interference effect varied across hand-approaching display conditions. The mean RT difference between the compatible and incompatible trials was significant in only the handshake condition, *t*(19) = 8.95, *p* < 0.001. In all control conditions, the mean RTs were not significantly different between the compatible and incompatible trials, *p*s > 0.12.

The same ANOVA was conducted for the accuracy data, revealing significant main effect of hand-approaching display, *F*(3,17) = 4.00, *p* < 0.05, $\eta_{p}^{2}$ = 0.41. It suggested that the accuracy varied with the background of the target and distractor. No other effect reached statistical significance, *F*s < 1.03, *p* > 0.32.

Thus, the handshake display showed its specificity in the interference task because of the significant interference effect. The possible reasons for the divergent results of the cueing and interference task are discussed in the General Discussion. Our results indicate that not all learned object associations lead to the attention effects induced by the handshake display; therefore, there must be something inherent in the handshake scene such that the attentional system treats the two hands as an inseparable unit. The key factor, we speculated, is likely to be the social information contained in it.

## General Discussion

This study demonstrates that, when two hands approach each other, seemingly to perform a handshake, the response to the target presented on one hand facilitated the attentional cue presented on the non-target hand (Experiment 1), and responses to a target on one hand was significantly impaired by a distractor on the other hand (Experiment 2). We confirmed that these effects were dependent on the existence of the hands, thus were due to modulating of the deployment of attention on the hands (Experiment 3). The findings were also not explained merely by perceptual familiarity or physical fit, because other paired objects that are common in daily life did not show the same effects (Experiment 4).

### The Social Nature of Attention Deployment

The handshake display contains several visual features and pieces of semantic information; hence, one might argue that the observed attention effects may not be the result of social factors. We posit, however, that the attention effects observed in the current experiments is associated with social factors for three reasons. First, similarity is not the cause of the attention effects. In the both-hand and both-palm conditions, the two hands displayed more similarities than those displayed in the handshake condition; yet, there was no such effect in the former two conditions. Second, the attention effects cannot be attributed to the impending action implied by the display, because all of the control conditions involved approach actions. Third, merely watching other peoples’ hands that are not socially related cannot lead to the attention effects. Taylor et al. (2015) showed that the attention shift across others’ hands is nothing special, but just like any other surface. Thus, in the current study, the social information brought by the handshake action should be the key factor to cause the attention effects. Fourth, mere perceptual familiarity and physical fit do not lead to exactly the same effects. Experiment 4 demonstrated that other familiar paired objects (such as hand and cup or plug and socket, which lock onto each other in a similar manner to hands) did not show the same pattern in modulating attention deployment—that is, the learned object associations facilitated the attentional shift from the cued object to the other, but the interference effect occurred only between the hands approaching to perform handshake. Thus, these results could not be solely explained by learned object associations; rather, the social information conveyed by the handshake action is likely to play an important role.

Based on the above reasons, we argue that the social relationship exhibited by the handshake scene leads to the attention effects observed in both the cueing and interference tasks, although we do not exclude the possibility that such pre-learned associations is needed for social information to induce such effects. Further studies are thus required to examine whether a novel social relationship between objects would lead to the same results.

Humans can make high-level cognitive inferences about the relationships between objects, intentionally regard them as a unit, and redeploy attention accordingly. Does the attention effect in mutual approaching hands, therefore, result from high-level cognitive inferences? We argue that it in fact occurs at a perceptual level. First, if the experimental manipulation were transparent to participants, then they might have intentionally employed strategies to modulate their responses. However, the hand-approaching display manipulation, and the task in the current experiments—to report the identity of the target—were ostensibly unrelated to the purpose of our experiments, which was to investigate how socially related objects modulate attention deployment. Thus, it was unlikely that the participants intended to respond according to experimenter expectations. Second, post-experiment interviews confirmed that participants failed to discern the actual purpose of the study. Indeed, the majority of participants believed that the hand-approaching display was employed to distract them and attempted to ignore it as a result. Thus, the effect occurred involuntarily, providing evidence that socially related objects do not affect attention deployment via higher-level cognitive processes.

### The Interaction between Social Processing and Attention Deployment

Converging with research in the gaze cueing effect (Frischen et al., 2007) and the attentional consequences induced by social interaction (Yin et al., 2013), the current findings provide further evidence that our visual system deploy attention according to social information. Such social-based attention deployment is probably achieved through the interaction between social and perceptual processes. Previous studies (Beck and Palmer, 2002; Kimchi and Hadad, 2002) have demonstrated that perceptual organization interacts with higher-level processes rather than working in a purely stimulus-driven, bottom-up fashion (Neisser, 1967; Beck, 1975; Marr, 1982; Treisman, 1986). In addition, Palmer et al. (2003) suggested that visual organization occurs at each level of representation rather than at a single level, and that the organization relies on features extracted from different levels. Thus, the interactive processes between the social and perceptual system enables organization according to various sources of visual information, including the social structure of a visual scene, such that attention could be deployed based on the perceptual units defined by the social relationship among objects.

Indeed, a growing body of research has indicated that vision is more social than expected. On the one hand, the visual system can recover social properties from the physical properties of objects and events. For example, although simple mechanical interactions between objects convey little information, the visual system can infer their social properties, producing strong impressions of animacy (Heider and Simmel, 1944; Scholl and Tremoulet, 2000; Schlottmann et al., 2006) and recovering social structures, such as causality, goals and intentions, from their interactions (Michotte, 1954/1963; Dasser et al., 1989; Dittrich and Lea, 1994; Gao et al., 2009, 2010). On the other hand, social information offers effective cues for lower-level perceptual processing. For example, a gaze cue guides our attention (Langton et al., 2000; Frischen et al., 2007), and changes in facial expressions influence the causal perceptions of physical events (Zhou et al., 2012). Furthermore, the present study revealed that even a lower-level perceptual process, such as attentional selection, is affected by social factors. Such effect is not unique to the handshake scene. Rather, it is more likely a common phenomenon that occurs in different social situations. Yin et al. (2013) demonstrated that a cooperative relationship, which was reflected in the dynamic chases, led to attentional consequence induced by the perceptual grouping of cooperative objects. In those cases, social information provides additional cues for the process through which people form effective inferences about the external world structure. More importantly, it reveals that social properties are assigned to visual representations early at the perceptual stages, suggesting that social processing begins to interact with visual perception at fundamental visual processes. The perceptual foundation of subsequent processing is thereby established to understand social structures and interact with other people. For example, if we observe two people talking to each other, we are likely to choose a path around them (rather than between them) when passing by. If these people are treated as a unit from the start at the perceptual stage and attention is deployed accordingly, then the cognitive system will plan a path around them without explicitly considering the social consequences of each possible path. Further studies should be conducted to examine the social-based attention effects in such real-life situations, applying the cognitive ethology approach (Kingstone et al., 2008; Kingstone, 2009), in order to generalize the experimental phenomena obtained from controlled lab situation to more stimulus-rich situations.

In addition, further studies are needed to investigate how social information modulate attention. Take the facilitated attentional shift in the handshake condition for an example, there are at least three possible mechanisms^1^ of this effect: First, the hands are perceptually grouped due to their social relationship, thus attention automatically spread within the perceptual group, leading to speeded attentional shift between two hands. Second, the handshake display speeds the disengagement of attention from one hand, therefore attention is easier to orient to the other hand. Third, the handshake display may “reset” the attention allocation to direct the focus to the middle of the display, resulting in faster responses in the invalid trials and slower responses in the valid trials, thus the cueing effect is weakened. Those possible accounts need to be tested to reveal the mechanisms of social-based attention modulation in follow-up research.

### Differences and Similarities between Social and Non-social Paired Objects

Experiment 4 revealed that the handshake display was similar to other non-social paired objects in facilitating attentional shift between objects—the response to the target present on one object was quicker when the other object was cued. However, they were different in the interference task, that is, the between-object interference occurred in only the handshake condition. This divergence is noteworthy, as it seems that the specificity of the social information included in the handshake display greatly reflects the interference effect.

It has been reported that the familiarity of the stimulus leads to the grouping of objects and consequently affects attention. For example, familiar words (Kumada and Humphreys, 2001) and action relationships (Humphreys and Riddoch, 2007) affect attentional selection, such that one attends to the related objects as a single unit. Our finding extends this line of research, suggesting that the learned social relationship between objects leads to a similar effect. The different behavior in the interference task suggests that it is more difficult to regard the socially related objects as two parts, leading to more difficulty in inhibiting the distractor on one of the objects, compared to the case of non-socially related objects. At least under the current experimental settings, the interference task may be more sensitive in detecting the specific attentional effect induced by socially related objects. Further study is needed to clarify what and how socially related objects differ from other familiar pairs in modulating attention.

## Conclusion

In summary, we demonstrated one example, the handshake-induced attention effects, as the evidence for the assumption that attention deployment can be modulated by the learned social relationships between objects. Generalization of such effects is required to further test this assumption, and to get more evidence to fully understand the role of social information in attention processes.

## Author Contributions

JZ, JY, and MS conceived and designed the experiments. JY and XD performed the experiments and collected the data. JY, JZ, and MS performed the data analyses. All authors contributed to the result interpretation and manuscript writing. All authors approved the final version of the manuscript for submission.

## Conflict of Interest Statement

The authors declare that the research was conducted in the absence of any commercial or financial relationships that could be construed as a potential conflict of interest.

## Footnotes

1 Thanks for a reviewer for pointing out the latter two possibilities.

## References

[B1] Baron-Cohen S. (1995). “The eye direction detector (EDD) and the shared attention mechanism (SAM): two cases for evolutionary psychology,” in *Joint Attention Its Origins and Role in Development*, eds Moore C. Dunham P. J. (Hillsdale, NJ: Lawrence Erlbaum Associates, Publishers), 41–59.

[B2] Beck D. M. Palmer S. E. (2002). Top-down influences on perceptual grouping. *J. Exp. Psychol. Hum. Percept. Perform.* 28 1071–1084. 12421056

[B3] Beck J. (1975). Relation between similarity grouping and perceptual constancy. *Am. J. Psychol.* 88 397–409. 10.2307/1421770 1200188

[B4] Bruzzo A. Borghi A. M. Ghirlanda S. (2008). Hand–object interaction in perspective. *Neurosci. Lett.* 441 61–65. 10.1016/j.neulet.2008.06.020 18599215

[B5] Chen Z. (1998). Switching attention within and between objects: the role of subjective organization. *Can. J. Exp. Psychol.* 52 7–17. 10.1037/h0087274

[B6] Cohen J. (1988). *Statistical Power Analysis for the Behavioral Sciences*, 2nd Edn Hillsdale, NJ: Lawrence Earlbaum Associates.

[B7] Dasser V. Ulbaek I. Premack D. (1989). The perception of intention. *Science* 243 365–367. 10.1126/science.2911746 2911746

[B8] Dittrich W. H. Lea S. E. (1994). Visual perception of intentional motion. *Perception* 23 253–268. 10.1068/p230253 7971105

[B9] Driver J. Davis G. Ricciardelli P. Kidd P. Maxwell E. Baron-Cohen S. (1999). Gaze perception triggers reflexive visuospatial orienting. *Vis. Cogn.* 6 509–540. 10.1080/135062899394920

[B10] Driver J. Davis G. Russell C. Turatto M. Freeman E. (2001). Segmentation, attention and phenomenal visual objects. *Cognition* 80 61–95. 10.1016/S0010-0277(00)00151-7 11245840

[B11] Duncan J. Duncan J. (1984). Selective attention and the organization of visual information. *J. Exp. Psychol. Gen.* 113 501–517. 10.1037/0096-3445.113.4.501 6240521

[B12] Egly R. Driver J. Rafal R. D. (1994). Shifting visual attention between objects and locations: evidence from normal and parietal lesion subjects. *J. Exp. Psychol. Gen.* 123 161–177. 10.1037/0096-3445.123.2.161 8014611

[B13] Faul F. Erdfelder E. Buchner A. Lang A.–G. (2009). Statistical power analyses using G*Power 3.1: tests for correlation and regression analyses. *Behav. Res. Methods* 41 1149–1160. 10.3758/BRM.41.4.1149 19897823

[B14] Faul F. Erdfelder E. Lang A.–G. Buchner A. (2007). G*Power 3: a flexible statistical power analysis program for the social, behavioral, and biomedical sciences. *Behav. Res. Methods* 39 175–191. 10.3758/BF03193146 17695343

[B15] Flombaum J. I. Scholl B. J. (2006). A temporal same-object advantage in the tunnel effect: facilitated change detection for persisting objects. *J. Exp. Psychol. Hum. Percept. Perform.* 32 840–853. 16846283 10.1037/0096-1523.32.4.840

[B16] Friesen C. K. Kingstone A. (1998). The eyes have it! Reflexive orienting is triggered by nonpredictive gaze. *Psychon. Bull. Rev.* 5 490–493. 10.3758/BF03208827

[B17] Friesen C. K. Ristic J. Kingstone A. (2004). Attentional effects of counterpredictive gaze and arrow cues. *J. Exp. Psychol. Hum. Percept. Perform.* 30 319–329. 15053691 10.1037/0096-1523.30.2.319

[B18] Frischen A. Bayliss A. P. Tipper S. P. (2007). Gaze cueing of attention: visual attention, social cognition, and individual differences. *Psychol. Bull.* 133 694–724. 10.1037/0033-2909.133.4.694 17592962 PMC1950440

[B19] Gao T. McCarthy G. Scholl B. J. (2010). The wolfpack effect: perception of animacy irresistibly influences interactive behavior. *Psychol. Sci.* 21 1845–1853. 10.1177/0956797610388814 21078895

[B20] Gao T. Newman G. E. Scholl B. J. (2009). The psychophysics of chasing: a case study in the perception of animacy. *Cogn. Psychol.* 59 154–179. 10.1016/j.cogpsych.2009.03.001 19500784

[B21] He X. Fan S. Zhou K. Chen L. (2004). Cue validity and object-based attention. *J. Cogn. Neurosci.* 16 1085–1097. 10.1162/0898929041502689 15298794

[B22] Heider F. Simmel M. (1944). An experimental study of apparent behavior. *Am. J. Psychol.* 57 243–259. 10.2307/1416950

[B23] Ho M. C. Atchley P. (2009). Perceptual load modulates object-based attention. *J. Exp. Psychol. Hum. Percept. Perform.* 35 166–11. 10.1037/a0016893 19968427

[B24] Humphreys G. W. Riddoch M. J. (2007). How to define an object: evidence from the effects of action on perception and attention. *Mind Lang.* 22 534–547. 10.1111/j.1468-0017.2007.00319.x

[B25] Kahneman D. Treisman A. Gibbs B. J. (1992). The reviewing of object files: object-specific integration of information. *Cogn. Psychol.* 24 175–219. 10.1016/0010-0285(92)90007-O 1582172

[B26] Kimchi R. Hadad B. S. (2002). Influence of past experience on perceptual grouping. *Psychol. Sci.* 13 41–47. 10.1111/1467-9280.00407 11892777

[B27] Kingstone A. (2009). Taking a real look at social attention. *Curr. Opin. Neurobiol.* 19 52–56. 10.1016/j.conb.2009.05.004 19481441

[B28] Kingstone A. Smilek D. Eastwood J. D. (2008). Cognitive ethology: a new approach for studying human cognition. *Br. J. Psychol.* 99 317–340. 10.1348/000712607X251243 17977481

[B29] Kumada T. Humphreys G. W. (2001). Lexical recovery on extinction: interactions between visual form and stored knowledge modulate visual selection. *Cogn. Neuropsychol.* 18 465–478. 10.1080/02643290042000224 20945225

[B30] Langton S. R. H. Watt R. J. Bruce V. (2000). Do the eyes have it? Cues to the direction of social attention. *Trends Cogn. Sci.* 4 50–59. 10.1016/S1364-6613(99)01436-9 10652522

[B31] Liuzza M. T. Setti A. Borghi A. M. (2012). Kids observing other kids’ hands: visuomotor priming in children. *Conscious. Cogn.* 21 383–392. 10.1016/j.concog.2011.09.015 22014465

[B32] Marr D. (1982). *Vision.* San Francisco, CA: Freeman.

[B33] Michotte A. (1954/1963). *The Perception of Causality* (trans. Miles T. R. Miles E.). London: Methuen.

[B34] Moore C. M. Yantis S. Vaughan B. (1998). Object-based visual selection: evidence from perceptual completion. *Psychol. Sci.* 9 104–110. 10.1111/1467-9280.00019

[B35] Neisser U. (1967). *Cognitive Psychology.* New York, NY: Appleton-Century-Crofts.

[B36] Palmer S. E. (2003). “Perceptual organization and grouping,” in *Perceptual Organization in Vision: Behavioral and Neural Perspectives*, eds Kimchi R. Behrmann M. Olson C. R. (Mahwah, NJ: Lawrence Erlbaum Associates), 3–43.

[B37] Palmer S. E. Brooks J. L. Nelson R. (2003). When does grouping happen? *Acta Psychol.* 114 311–330. 10.1016/j.actpsy.2003.06.003 14670702

[B38] Reed C. L. Grubb J. D. Steele C. (2006). Hands up: attentional prioritization of space near the hand. *J. Exp. Psychol. Hum. Percept. Perform.* 32 166–177. 16478334 10.1037/0096-1523.32.1.166

[B39] Sato W. Okada T. Toichi M. (2007). Attentional shift by gaze is triggered without awareness. *Exp. Brain Res.* 183 87–94. 10.1007/s00221-007-1025-x 17624520

[B40] Schlottmann A. Ray E. D. Mitchell A. Demetriou N. (2006). Perceived physical and social causality in animated motions: spontaneous reports and ratings. *Acta Psychol.* 123 112–143. 10.1016/j.actpsy.2006.05.006 16908002

[B41] Scholl B. J. (2001). Objects and attention: the state of the art. *Cognition* 80 1–46. 10.1016/S0010-0277(00)00152-9 11245838

[B42] Scholl B. J. Tremoulet P. D. (2000). Perceptual causality and animacy. *Trends Cogn. Sci.* 4 299–309. 10.1016/S1364-6613(00)01506-0 10904254

[B43] Sternberg R. J. (2003). *Cognitive Psychology*, 3rd Edn Belmont, CA: Thomson Wadsworth.

[B44] Taylor J. E. T. Pratt J. Witt J. K. (2015). Joint attention for stimuli on the hands: ownership matters. *Front. Psychol.* 6:543 10.3389/fpsyg.2015.00543 PMC4416455 25983713

[B45] Taylor J. E. T. Witt J. K. (2014). Altered attention for stimuli on the hands. *Cognition* 133 211–225. 10.1016/j.cognition.2014.06.019 25051509

[B46] Treisman A. (1986). “Properties, parts, and objects,” in *Handbook of Perception and Human Performance* Vol. 2 eds Boff K. R. Kaufman L. Thomas J. P. (New York, NY: Wiley), 1–70.

[B47] Wertheimer M. (1923). Untersuchungen zur Lehre von der Gestalt. II. *Psychol. Res.* 4 301–350. 10.1007/BF00410640

[B48] Yin J. Ding X. Zhou J. Shui R. Li X. Shen M. (2013). Social grouping: perceptual grouping of objects by cooperative but not competitive relationships in dynamic chase. *Cognition* 129 194–204. 10.1016/j.cognition.2013.06.013 23896212

[B49] Zhou J. Huang X. Jin X. Liang J. Shui R. Shen M. (2012). Perceived causalities of physical events are influenced by social cues. *J. Exp. Psychol. Hum. Percept. Perform.* 38 1465–1475. 10.1037/a0027976 22506780

